# Web-Based Prediction Models for Overall Survival and Cancer-Specific Survival of Patients With Primary Urachal Carcinoma: A Study Based on SEER Database

**DOI:** 10.3389/fpubh.2022.870920

**Published:** 2022-06-02

**Authors:** Li Ding, Bin Xia, Yang Zhang, Zijie Liu, Junqi Wang

**Affiliations:** Department of Urology, The Affiliated Hospital of Xuzhou Medical University, Xuzhou, China

**Keywords:** urachal carcinoma, prognostic factors, SEER, validation, urogenital malignancies, survival analysis, nomogram

## Abstract

**Objective:**

We aimed to establish nomograms to predict the overall survival (OS) and cancer-specific survival (CSS) of patients with primary urachal carcinoma (UrC).

**Methods:**

Information on patients diagnosed with UrC from 1975 to 2018 was collected from the Surveillance, Epidemiology, and End Results (SEER) Program Research Data. The independent prognostic factors were determined using univariate and multivariate Cox regression. Backward variable elimination according to the Akaike information criterion (AIC) identified the most accurate and parsimonious model. Nomograms were built based on regression coefficients. The C-index, calibration plot, Brier score, integrated discrimination improvement (IDI), area under the receiver operating curve (AUC), and decision curve analysis (DCA) curve were used to evaluate the efficiency of models.

**Results:**

In total, 236 patients obtained from SEER were divided randomly into training and validation cohorts in a 70:30 ratio (166 and 70 patients, respectively). In the training cohort, multivariate Cox regression analysis indicated that pTNM/Sheldon/Mayo staging systems (included respectively), age, and tumor grade were independent prognostic factors for OS. A similar result was also found in CSS. While other variables, such as radiotherapy and chemotherapy, did not identify significant correlations. In predicting OS and CSS at 3- and 5- years, the nomograms based on pTNM showed superior discriminative and calibration capabilities in comparison to multiple statistical tools. The C-index values for the training cohort were 0.770 for OS and 0.806 for CSS, and similar outcomes were shown in further internal validation (C-index 0.693 for OS and 0.719 for CSS). We also discovered that the link between age at diagnosis and survival follows a U-shaped curve, indicating that the risk of poor prognosis decreases first and then increases with age.

**Conclusion:**

The efficacy of pTNM in predicting the prognosis of patients with UrC was greater than that of the Sheldon and Mayo staging system. Therefore, we recommend pTNM as the preferred system to stage UrC. The novel constructed nomograms based on pTNM, age, and tumor grade showed high accuracy and specificity and could be applied clinically to predict the prognosis of patients with UrC.

## Introduction

Urachal carcinoma (UrC) is a rare but highly malignant neoplasm arising from a urachal remnant, which is located between the umbilicus and the dome of the bladder (1–3). It is a rare type of bladder tumor, and it is characterized by high malignancy, late stage, and poor prognosis, accounting for only 0.01% of all adult malignancies and 0.2–0.5% of all bladder cancers (3–6). The annual incidence of UrC in the general population is estimated to be 1 in 5 million (7). Adenocarcinoma is the most prevalent histologic type of urachal cancer, which accounts for ~90% of the cases in the Mayo Clinic series. Mucinous adenocarcinoma is the most common subtype of adenocarcinoma, followed by signet ring cell shape and adenocarcinoma not otherwise specified (NOS). Other histological types, such as urothelial carcinomas, squamous cell carcinomas, sarcomas, and undifferentiated carcinomas have also been reported (8–10). Surgery is the only known way to treat UrC, and based on urachal adenocarcinoma or non-urachal adenocarcinoma, two surgical options, partial and radical cystectomy, are available. Partial patients received a combination of bladder drug instillation, chemotherapy, and/or radiation therapy following surgery.

Unfortunately, there is no validated staging system for UrC. Different staging approaches have been described, namely, Sheldon, Mayo, and modified TNM staging systems (7, 10, 11). In searching the Surveillance, Epidemiology, and End Results (SEER) database, we also found SEER's specific staging approach: the historic SEER stage A in the past. Sheldon was first proposed in 1984, and it remains the most clinically used system, although it has never been officially validated. The study from Mayo Clinic concluded that the Sheldon staging system is too cumbersome and over-specified, while the Mayo system was found to be more balanced in terms of UrC patient distribution. However, a second team from Mayo Clinic chose to use the more general TNM staging system. The indiscriminate use of multiple different systems is not only detrimental to the establishment of an international unified standard but also increases the difficulty of clinical research on UrC in subsequent studies, while the widespread use of the Sheldon system, whose accuracy is still unknown, may result in a portion of patients with potential poor prognostic risk not receiving timely clinical management. Therefore, comparing the efficacy of the three systems and, through comparison, selecting the one with the best predictive ability as a unified clinical consensus is necessary to benefit patients and can reduce unnecessary clinical disagreements. It is critical to find prognostic variables in patients with UrC to predict survival and perform a clinical evaluation. Several prior studies have shown predictive factors for patients with UrC, such as tumor stage, positive intraoperative margins, and pathological grade, whether positive lymph nodes were found in lymph node dissection, and the surgical approach performed (7, 10, 12–14). Due to the small sample size and non-unique staging criteria, however, the prediction value of the various indicators is restricted. As a result, a large case series and unified staging standards should be used to uncover key prognostic markers in patients with UrC, and a precise forecasting method should be built.

We are committed to building a user-friendly and meaningful statistical prediction model to determine the prognosis for UrC. A nomogram is a prediction model that can intuitively predict the probability of an event and is currently widely used in the prediction of clinical efficacy of various diseases (15–17). According to the inclusion of each variable in the line graph, the scores corresponding to each predictor can be obtained, and the scores are summarized and recorded in the total score, which corresponds to the survival probability of the patient. This visual predictive tool has great potential for clinical applications, and the nomogram-based online web calculator is extremely convenient for clinicians to use. Nonetheless, as we now know, due to the rarity of the disease, prognostic models for patients with UrC have been rarely studied. We wanted to incorporate and analyze clinicopathological features that may be related to the prognosis of patients with UrC based on the SEER database, and select appropriate factors to construct a nomogram to predict the survival prognosis of patients with UrC.

## Materials and Methods

### Patients and Factors

A retrospective cohort research approach was adopted. The information came from the SEER Program Research Data, which covers approximately 27.8% of the United States population. We used ICD-O-3 site codes C67.7 and histological codes 8140–8147 and 8255–8490 to identify UrC. The inclusion factors included: survival time, survival outcomes, age of diagnosis, gender, race, histologic type, tumor grade, TNM stage, surgical procedures, and lymphadenectomy/radiotherapy/chemotherapy data. Using the obtained TNM system, we re-staged all patients with the Sheldon and Mayo staging systems. Overall survival (OS) was defined as survival until death from any cause, while cancer-specific survival (CSS) was defined as survival until death due to UrC.

### Statistical Analysis

Cancer-specific survival was the primary endpoint, whereas OS was the secondary endpoint. In a random 70:30 split ratio, the primary SEER cohort was split into training and internal validation cohorts. All the data were summarized as percentages of categorical variables, and chi-square tests were used to compare them. The Kaplan–Meier method was used to determine the clinical endpoints of the patients, and the log-rank test was used to analyze them. To assess the impact of the variables on OS and CSS, a backward stepwise (according to the Akaike information criterion [AIC]) multivariate Cox proportion analysis was performed. Hazard ratios (*HR*s) estimated from the Cox analysis were mentioned as relative risks (*RR*s) with corresponding 95% confidence intervals (*CI*s). For predicting 3/5-year OS and CSS, nomograms based on multivariate models were constructed and verified. In both the training and validation cohorts, the C-index was employed to measure the improved model's discrimination. The optimal risk total points cut-off was calculated using the X-tile software. Accuracy and benefits were evaluated by comparing the nomograms with the area under the receiver operating curve (AUC), integrated discrimination improvement (DCA), and integrated discrimination improvement (IDI) (18). Brier scores vary between 0 and 1, where a lower Brier score is indicative of a better-calibrated prediction (19). X-tile, SPSS 26.0, and R 4.1.2 were used to statistically investigate the clinical information of patients with UrC. Only a two-sided value of *p* < 0.05 was considered statistical significance.

## Results

### Patient Characteristics

Between 1975 and 2018, 501 patients diagnosed with UrC as their initial tumor met the initial inclusion criteria. The remaining 236 individuals were included in the research after 265 patients were eliminated owing to ambiguous data details. In Supplementary Figure S1 and Supplementary Table S1, the procedure of data selection and the characteristics of patients accessible from the SEER database are shown. In the SEER database, the mean follow-up time was 69.28 months, and the median follow-up time was 58.5 months. The 3-year OS rate was (65.0 ± 3.1)%, and the 5-year OS rate was (55.4 ± 3.4)%. The 3-year CSS rate was (71.6 ± 3.0)%, and the 5-year CSS rate was (65.0 ± 3.4)%. The majority of patients were between the ages of 45 and 74 years (66.1%). The majority of patients were men (58.1%). The majority of patients were white (79.2%). Adenocarcinoma (such as not otherwise specified (NOS) and mucinous adenocarcinoma) is the most common histologic type of UrC (71.2%). The majority of patients were in tumor grades I and II (65.3%), which we considered to be well graded. The most commonly performed surgical procedure was partial cystectomy (69.5%). We included in our analysis the possible prognostic impact of interventions other than surgery of the primary lesion, and according to the analysis, it was known that just a few patients received lymphadenectomy (42.4%), chemotherapy (24.6%), and/or radiotherapy (6.4%) following surgery. After data validation and re-rating, most subjects were in the pT3/N0/M0 and Sheldon III/Mayo II stages. In our preliminary statistical treatment, SEER historic stage A was found to have a significantly lower predictive value than the three systems discussed above, so it is not discussed in this article. In both the training and validation cohorts, the distribution proportions of the subgroups were similar to the total set above.

### Construction of the Nomograms

**Figure 1:**
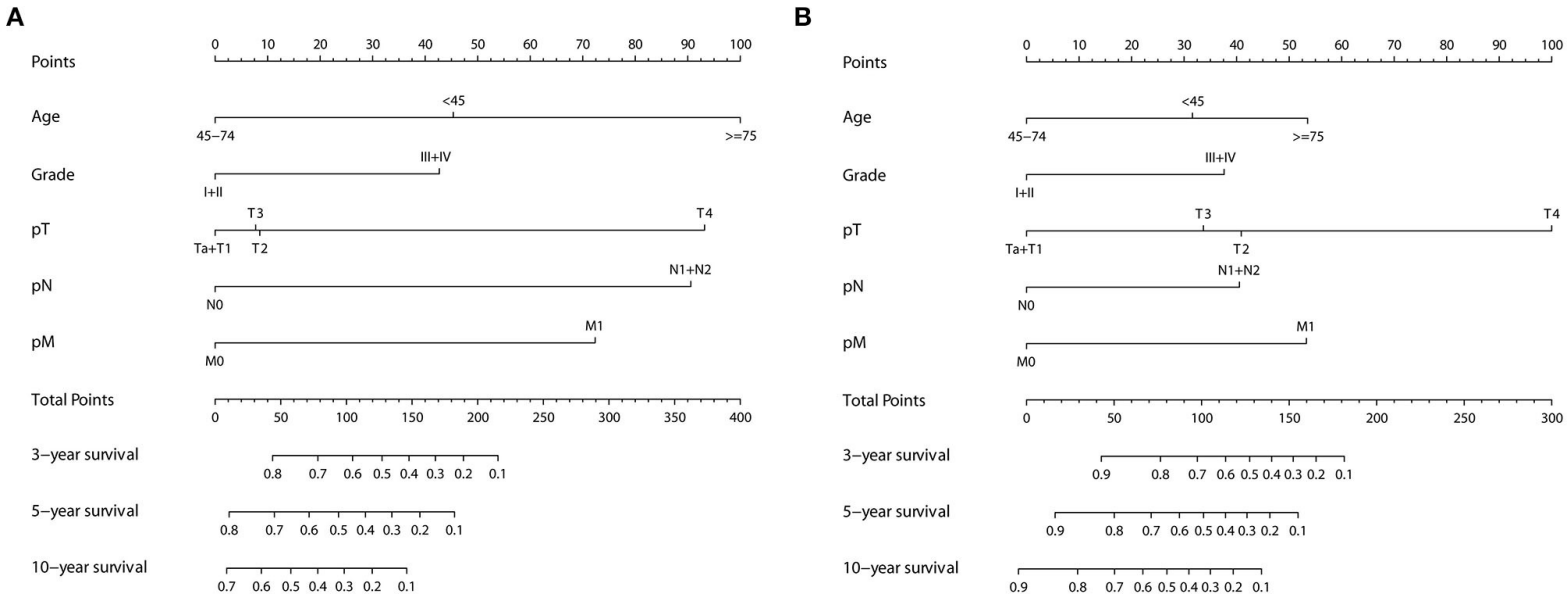
Nomogram predicting the 3-, 5-, and 10-years **(A)** OS and **(B)** CSS of patients with primary urachal carcinoma by summing the points identified on the points scale for each variable. The total points projected on the bottom scales determine the probability of survival.

In univariate Cox regression, as shown in Supplementary Figures S2, S3, age, tumor grade, pTNM, and Sheldon and Mayo staging systems were all independently associated with both OS and CSS (all *p* < 0.001). Given the collinearity among three staging variables, three predictors were fitted into different multivariable models (model 1 = age + grade + pTNM, model 2 = age + Sheldon, and model 3 = age + Mayo for OS; model 1 = age + grade + pTNM, model 2 = age + grade + Sheldon, and model 3 = age + grade + Mayo for CSS). The variables with *p* < 0.05 in the univariate analysis were assessed by using the multivariate Cox regression, utilizing a backward elimination procedure to define the independent prognostic indicators and estimate their influence on OS and CSS for patients in the training cohort. After backward stepwise regression analysis (according to the AIC), tumor grade in Sheldon (*p* = 0.102) and Mayo (*p* = 0.089) for OS were removed, and all other factors included in the multivariate Cox regression turned out to be essential in their own groups (Tables 1, 2). The nomograms based on the pTNM for OS and CSS prediction were constructed using these selected indicators (Figure 1). We may deduce from the nomograms that age had a significant influence on both OS and CSS. We also discovered that the link between age at diagnosis and survival is non-linear and follows a U-shaped curve, implying that patients who were diagnosed before 45 and beyond 75 years old had a poorer prognosis than those who were diagnosed between 45 and 75 (the median is 60) years old, which is similar to the finding of Yu et al. (17). After analysis and comparison, web-based versions of our nomograms are available at https://dl0710.shinyapps.io/UrC_OS/ and https://dl0710.shinyapps.io/UrC_CSS/, which can be conveniently applied to the clinical assessment of the prognostic risk of patients with UrC.

**Table 1:** Multivariate Cox analyses for the prediction of overall survival with urachal carcinoma.

	**Model 1**		**Model 2**		**Model 3**	
	**HR (95%CI)**	***P* value**	**HR (95%CI)**	***P* value**	**HR (95%CI)**	***P* value**
**Age (year)**		<0.001		<0.001		<0.001
<45	1 (referent)		1 (referent)		1 (referent)	
45–74	0.541 (0.316–0.925)		0.546 (0.321–0.929)		0.545 (0.320–0.928)	
≥75	2.069 (1.022–4.189		2.851 (1.444–5.627)		2.862 (1.449–5.651)	
**Grade**		0.033				
I + II	1 (referent)					
III + IV	1.774 (1.048–3.004)					
**pT**		0.004				
Ta + T1	1 (referent)					
T2	1.127 (0.511–2.484)					
T3	1.110 (0.546–2.258)					
T4	3.507 (1.517–8.108)					
**pN**						
N0	1 (referent)					
N1+N2	3.407 (1.651–7.028)					
**pM**						
M0	1 (referent)					
M1	2.637 (1.263–5.508)					
**Sheldon**				<0.001		
I			1 (referent)			
II			1.140 (0.480–2.707)			
III			1.524 (0.744–3.122)			
IV			7.797 (3.478–17.480)			
**Mayo**						<0.001
I					1 (referent)	
II					1.425 (0.816–2.490)	
III					7.700 (3.269–18.134)	
IV					7.043 (3.411–14.540)	

*HR, hazard ratio; CI, confidence interval; model 1 = age+grade+pTNM; model 2 = age+Sheldon; model 3 = age + Mayo*.

**Table 2:** Multivariate Cox analyses for the prediction of cancer-specific survival with urachal carcinoma.

	**Model 1**		**Model 2**		**Model 3**	
	**HR (95%CI)**	***P* value**	**HR(95%CI)**	***P* value**	**HR(95%CI)**	***P* value**
**Age (year)**		0.004		<0.001		<0.001
<45	1 (referent)		1 (referent)		1 (referent)	
45–74	0.497 (0.273–0.906)		0.510 (0.280–0.928)		0.516 (0.283–0.939)	
≥75	1.612 (0.689–3.72)		2.418 (1.091–5.363)		2.405 (1.084–5.336)	
**Grade**		0.008		0.031		0.021
I + II	1 (referent)		1 (referent)		1 (referent)	
III + IV	2.287 (1.247–4.196)		1.824 (1.057–3.148)		1.908 (1.101–3.305)	
**pT**		<0.001				
Ta + T1	1 (referent)					
T2	2.482 (0.799–7.711)					
T3	2.104 (0.713–6.208)					
T4	9.136 (2.850–29.284)					
**pN**		0.037				
N0	1 (referent)					
N1 + N2	2.477 (1.055–5.817)					
**pM**		0.003				
M0	1 (referent)					
M1	3.229 (1.492–6.986)					
**Sheldon**				<0.001		
I			1 (referent)			
II			2.908 (0.780–10.836)			
III			3.978 (1.197–13.223)			
IV			19.002 (5.34–67.614)			
**Mayo**						<0.001
I					1 (referent)	
II					2.023 (1.014–4.036)	
III					7.716 (2.605–22.849)	
IV					10.301 (4.471–23.734)	

*HR, hazard ratio; CI, confidence interval; model 1 = age + grade + pTNM; model 2 = age + grade + Sheldon; model 3 = age + grade + Mayo*.

### Verification of the Nomograms

**Figure 2:**
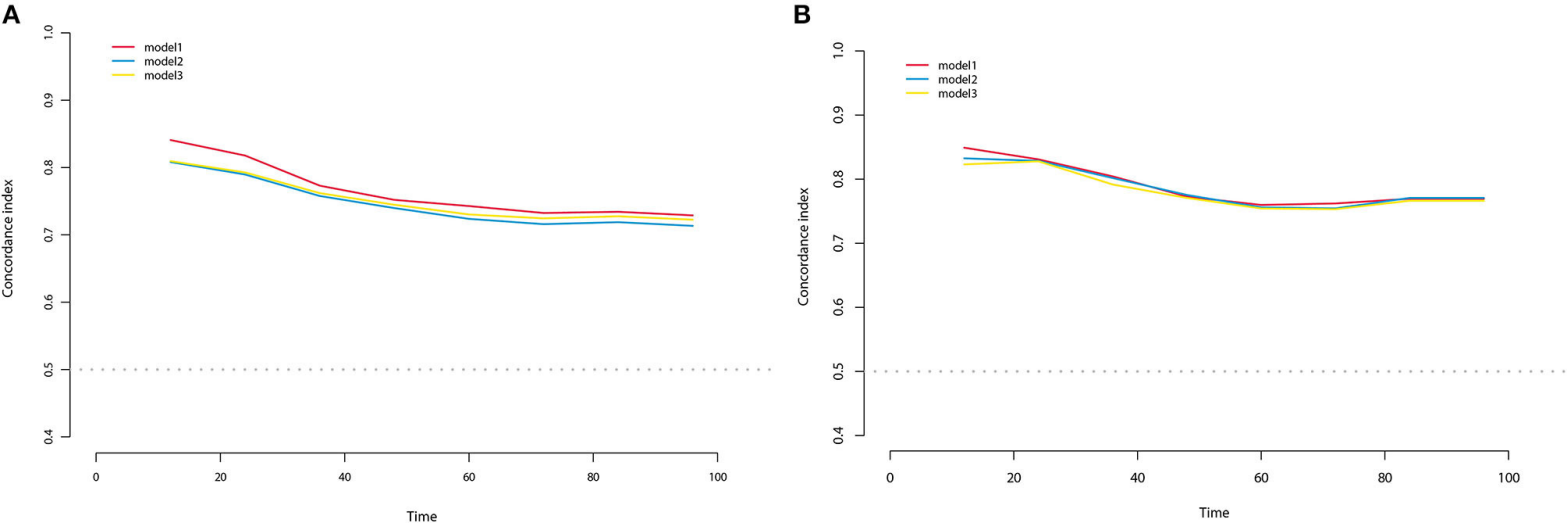
C-index for OS **(A)** and CSS **(B)** at different time of three nomogram models in the training cohort.

**Figure 3:**
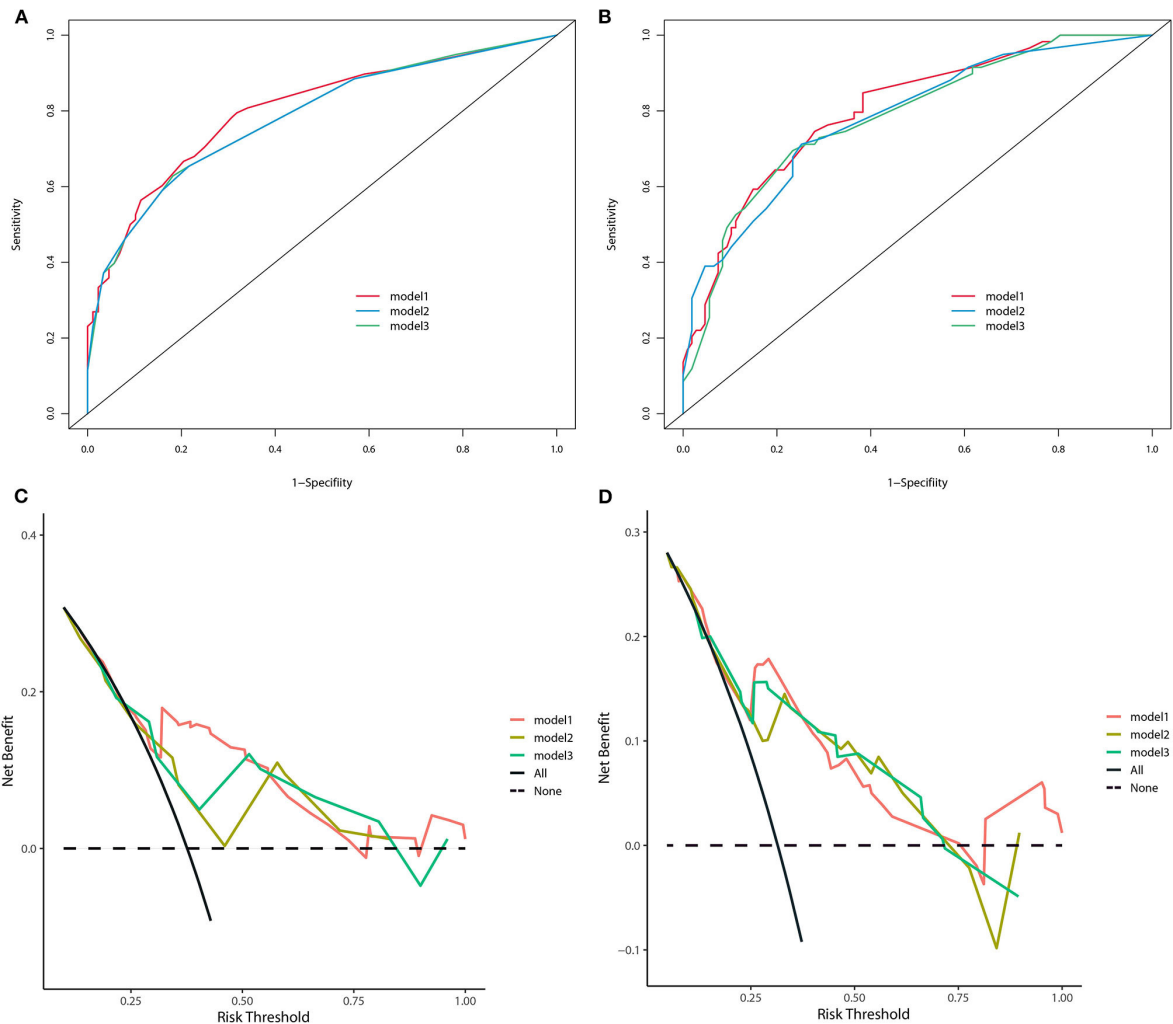
The receiver operating curve of three nomogram models in the training cohort **(A)** for OS and **(B)** for CSS. Decision-curve analyses demonstrating the net benefit associated with the use of the models **(C)** for OS and **(D)** for CSS.

All three staging systems showed good calibration and discrimination abilities. The time variable C-index of three models is listed in Figure 2, and model 1, with pTNM included, yielded the highest C-index. Model 2 (C-index 0.729, 95% *CI* 0.670–0.788, *p* < 0.001) and model 3 (C-index 0.736, 95% *CI* 0.678–0.793, *p* < 0.001) predicted OS with good discrimination, but model 1 (C-index 0.770, 95% *CI* 0.715–0.825, *p* < 0.001) predicted it more powerfully. The same conclusion was reached when predicting CSS with model 1 (C-index 0.806; 95% *CI* 0.755–0.857, *p* < 0.001). The 0.199 Brier score of model 1 indicated that the nomogram had higher accuracy, compared with model 2 (0.218) and model 3 (0.210). Among these models, model 1 yielded the highest AUC of 0.801 (95% *CI* 0.734–0.869) for OS and 0.784 (95% *CI* 0.712–0.855) for CSS (Figure 3). In Figure 3, which depicts the prediction of OS and CSS, the application of model 1 resulted in the highest net benefit. In the group inter-comparison among the three models, model 1 showed an improvement relative to the other two models (model 1 vs. model 2, IDI = 5.4%; model 1 vs. model 3, IDI = 2.9%) for OS. Based on the comparison of the available statistical analysis results, model 1 (such as pTNM, age, and tumor grade) was selected as the best model for further analyses.

**Figure 4:**
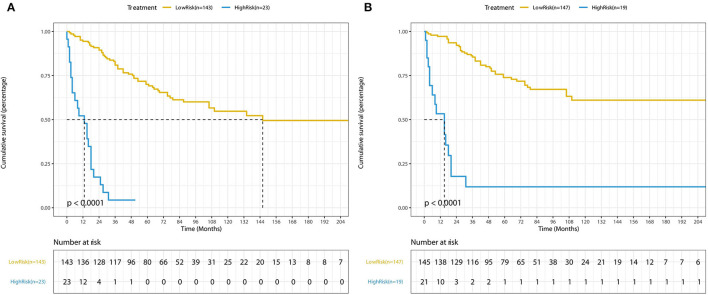
Kaplan-Meier curves of low-risk and high-risk based on the predictions of the nomograms **(A)** for OS and **(B)** for CSS.

**Figure 5:**
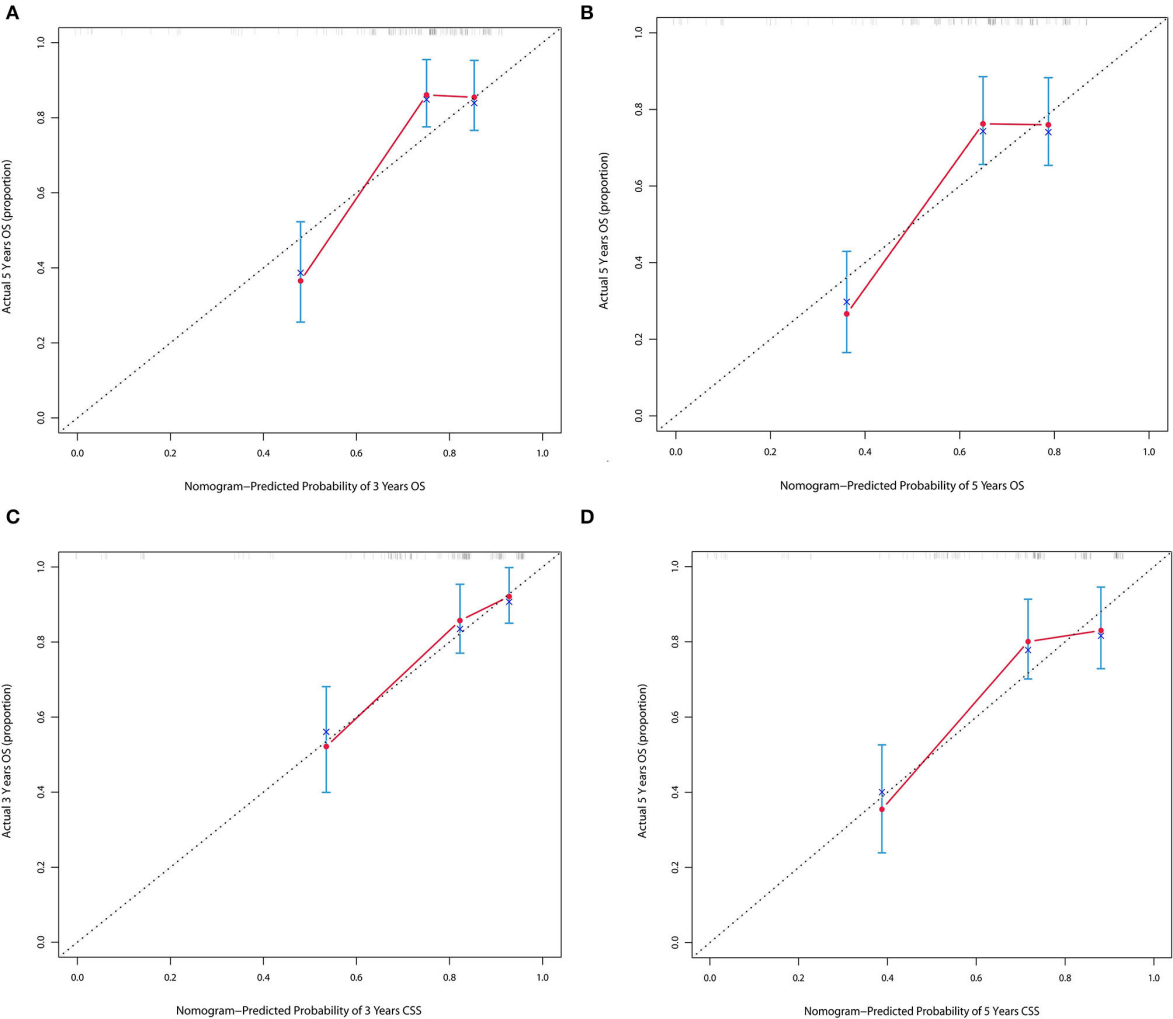
**(A,B)** Calibration plot of nomograms in 3- and 5-years in the training cohort for OS. **(C,D)** Calibration plot of nomograms in 3- and 5-years in the training cohort for CSS.

**Figure 6:**
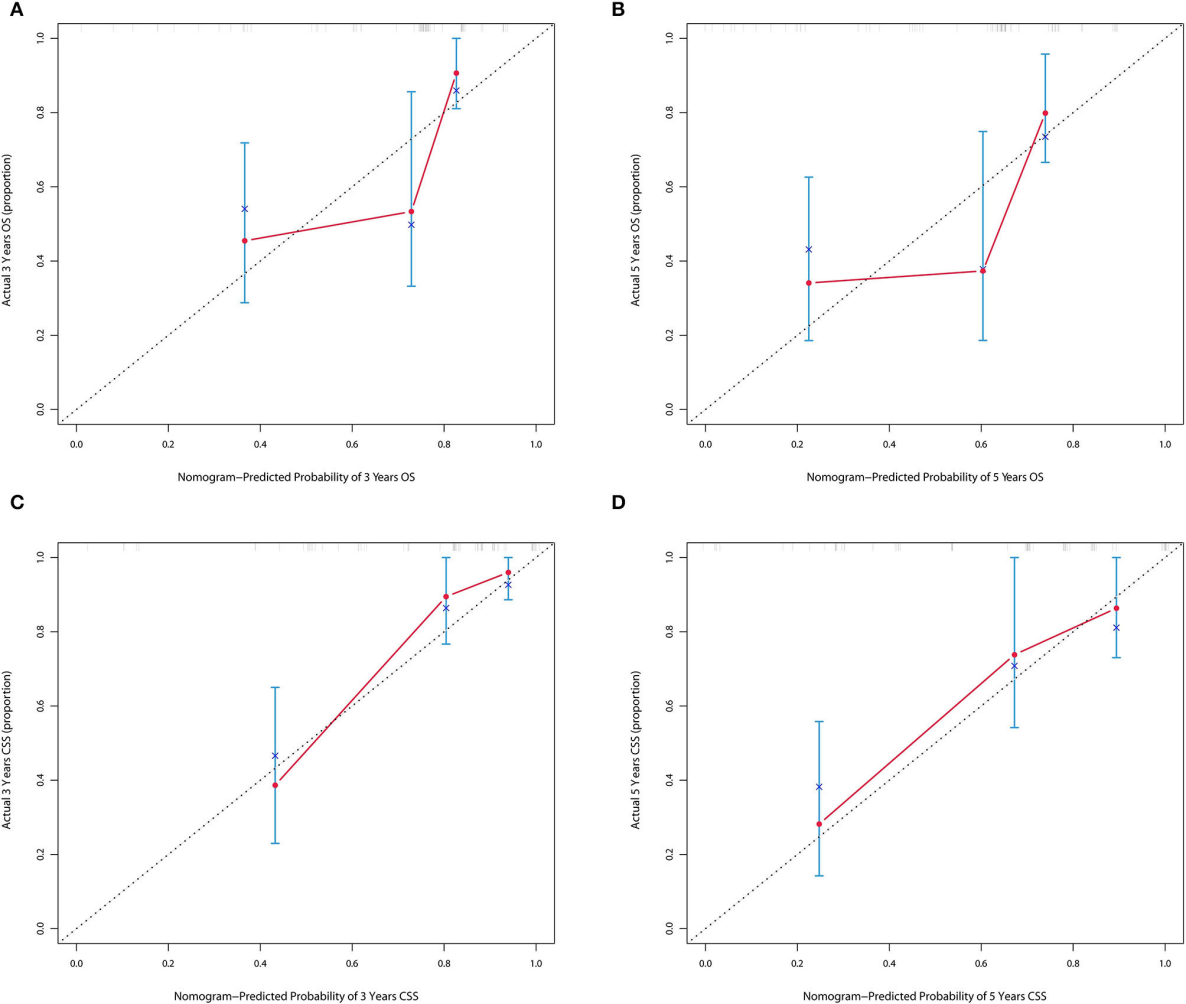
**(A,B)** Calibration plot of nomograms in 3- and 5-years in the validation cohort for OS. **(C,D)** Calibration plot of nomograms in 3- and 5-years in the validation cohort for CSS.

The forecasted OS and CSS may be calculated by adding the total scores of the factors that were considered. We divided patients into low- and high-risk groups based on various cutoff values (142.7 for OS and 131.6 for CSS) of the total points obtained by the X-tile software and used a Kaplan–Meier survival analysis to estimate their survival. In the training cohort, patients in the high-risk group had clearly shorter OS and CSS (both *p* < 0.0001) than patients in the low-risk group (Figure 4). The mean OS times were 142 and 13 months in the low-risk (*n* = 143) and high-risk (*n* = 23) groups, respectively. Meanwhile, the median CSS times were 159 and 36 months in the low-risk (*n* = 147) and high-risk (*n* = 19) groups, respectively. Additionally, the calibration plot for the likelihood of 3- and 5-year OS and CSS was close to the reference line, indicating that predictions and observations were in fair agreement. The C-index values for the training dataset were 0.770 for OS and 0.806 for CSS, and the internal validation cohort's C-index for predicting OS and CSS was 0.693 (95% *CI* 0.597–0.789) and 0.719 (95% *CI* 0.618–0.820) (see Figures 5, 6).

## Discussion

The urachus is a fibrous remnant of the allantois. After birth, it remains throughout life as the medial umbilical ligament running from the apex of the bladder to the umbilicus but without any further physiological role (3). UrC most often forms in the patent urachal duct (20). The annual incidence of this malignancy in the general population is estimated to be 1 in 5 million (7). Due to its rarity, targeted studies are mostly single-centered and small samples. As a rare malignant tumor of the genitourinary system, UrC is characterized by atypical clinical symptoms, bleak prognoses, and rapid disease progression (3, 5). Although UrC is included as a category of bladder malignancy, previous studies have shown that the two should not be confused in clinical management (21). Due to the lack of typical clinical symptoms of the disease, the difficulty in identifying the disease even with cystoscopy, and the lack of a clear consensus on the choice of comprehensive treatment, the disease is often advanced at the time of presentation, leading to a poor prognosis (22–24). In approximately 80% of patients, macroscopic or microscopic hematuria is a common medical manifestation, indicating that cancer has transgressed the muscularis mucosae and infiltrated the urothelial surface (9). Other uncommon clinical signs include urinary tract infections, umbilical infections, painful urination, and periumbilical masses (10). Some patients develop distant metastases even after surgery, such as liver, lung, bone, pelvic, and retroperitoneal lymph nodes, and there are no accepted data describing the probability of metastases to different organs and tissues (9, 25). Existing immunology and molecular biology studies have discovered a link between umbilical ductal carcinoma and gastrointestinal cancers, such as CD7 and KRAS (26, 27).

In the absence of an accepted and accurate grading system, modified TNM and Sheldon and Mayo staging systems are all clinically adopted (7, 9, 10). The Sheldon system was the first staging system to be detailed, and it is still the most often used, despite never having been formally confirmed. The Mayo system was created based on 49 patients with the diagnosis of urachal cancer who were seen at the Mayo Clinic. They are both simplified based on TNM. The criteria for the Sheldon system were as follows: Sheldon I: Ta–T1 and N0 and M0; Sheldon II: T2 and N0 and M0; Sheldon III: T3–T4 and N0 and M0; and Sheldon IV: T3–T4 and/or N1 and/or M1. The criteria for Mayo staging were as follows: Mayo I: Ta–T2 and N0 and M0; Mayo II: T3–T4 and N0 and M0; Mayo III: any T and N1 and M0; Mayo IV: any T and N2, and/or M1. In addition to the three staging systems mentioned above, the SEER database has its own staging system named SEER historic stage A, which grades UrC as localized, regional, and distant. Furthermore, we included SEER historic stage A in our statistical analysis, but since its efficacy is significantly lower than the above three, we do not dwell on it in this article. In addition to surgical resection of the local lesion and bladder, a variety of therapies, such as intravesical instillation, radiation, chemotherapy, and targeted therapies, are also used in the management of patients with UrC. However, the effectiveness of these measures cannot be evaluated yet. Therefore, we purposely requested the SEER Plus database, which contains radiotherapy data, for data extraction.

In our current study, age at diagnosis, histologic grading, and all three staging systems were shown to be significantly associated with OS and CSS. By applying different model comparison tools, we concluded that the pTNM staging system exhibited higher accuracy compared with the Sheldon and Mayo stages. The nomograms based on pTNM and two other influencing factors also showed good agreement in both the randomly obtained internal validation cohorts. In all the nomograms created, we can observe a risk inflection point at an age of around 60 years. The poor prognosis of elderly patients is to be expected, considering that their general condition is usually poor and they may have underlying diseases, such as hypertension and diabetes mellitus that prevent them from tolerating surgery or other treatment options. In contrast, the bleak prognosis in young people may be related to the biology of UrC, and further studies in the fields of pathology and molecular biology are needed in this group of patients. Meanwhile, our findings may lead clinicians to focus more on the clinical management of young patients. Based on our analysis, we believe that adjuvant treatment modalities such as radiotherapy and chemotherapy have not yet proven to be effective for UrC. The comprehensive treatment of patients with UrC after surgery still needs to be further investigated. Our study is not devoid of limitations. First, although the data we used for our modeling came from a large public dataset, it is important to acknowledge the inherent bias of this retrospective registry-based study. Information on several promising variables, such as underlying disease history, serologic indicators, individualized chemotherapy regimens used, and immunohistochemistry, was not available. Second, the Sheldon and Mayo categories were both re-staged by us because they were not directly available. Then again, the practical application of the model obtained based on the predominantly Caucasian SEER database for other centers, such as China, is unknown due to inevitable ethnic differences across countries and regions. More validations with a wider external sample size are needed to corroborate our findings before they can be used generally. Nonetheless, our research is a significant step toward developing predictive nomograms for predicting the prognosis of patients with UrC.

## Conclusion

In this study, we developed and validated nomograms and built online calculators for effective prediction of OS and CSS in UrC by comparing three clinically applied staging systems. We suggest using the nomograms based on the TNM staging system because they have better predictive efficacy. Our nomogram-based web calculators have great clinical applicability and can help the clinic individualize clinical assistance to patients.

## Data Availability Statement

The original contributions presented in the study are included in the article/Supplementary Material, further inquiries can be directed to the corresponding author/s.

## Author Contributions

LD, BX, YZ, and ZL contributed to the idea and design. BX collected and analyzed the data. LD, YZ, and ZL drew the figures and tables. LD and BX wrote the draft. LD, BX, YZ, ZL, and JW contributed to manuscript writing and revision. All authors approved the final manuscript.

## Funding

This study was sponsored by the Second Round of Xuzhou Medical Leading Talents Training Project (No. XWRCHT20210027).

## Conflict of Interest

The authors declare that the research was conducted in the absence of any commercial or financial relationships that could be construed as a potential conflict of interest.

## Publisher's Note

All claims expressed in this article are solely those of the authors and do not necessarily represent those of their affiliated organizations, or those of the publisher, the editors and the reviewers. Any product that may be evaluated in this article, or claim that may be made by its manufacturer, is not guaranteed or endorsed by the publisher.

## Supplementary Material

Supplementary Table S1 Baseline characteristics of patients with UrC in the SEER database.

Supplementary Figure S1 Flowchart for patient selection of the SEER database.

Supplementary Figure S2 The Forest plot of univariable Cox regression analysis of training cohort for predicting OS.

Supplementary Figure S3 The Forest plot of univariable Cox regression analysis of training cohort for predicting CSS.
