# Supplementary figures and images for: Web-Based Prediction Models for Overall Survival and Cancer-Specific Survival of Patients With Primary Urachal Carcinoma: A Study Based on SEER Database
Source: Front Public Health. 2022 Jun 2;10:870920. doi: 10.3389/fpubh.2022.870920 (PMC9201252; doi:10.3389/fpubh.2022.870920)

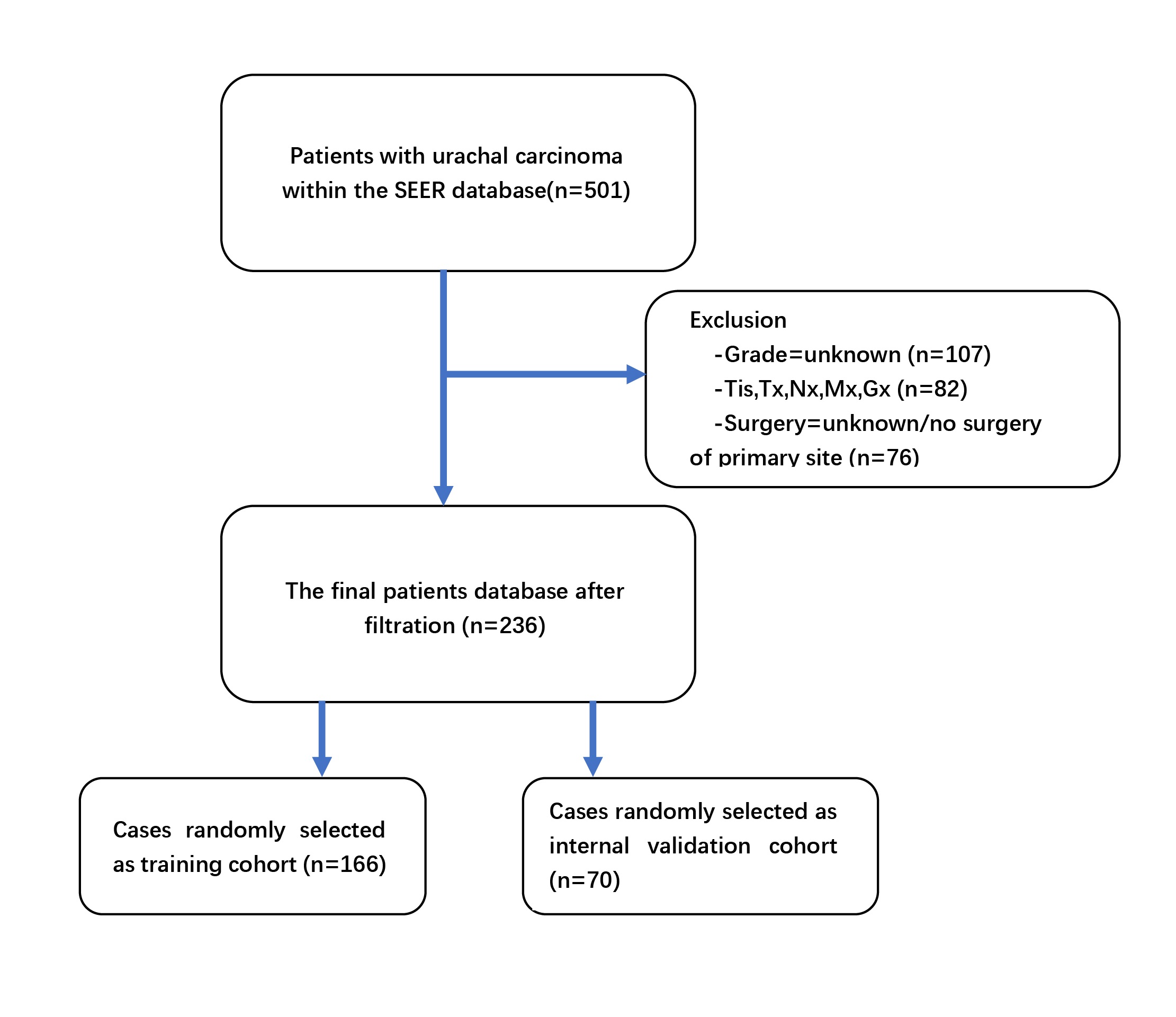

Supplement: Supplementary Figure S1 — Flowchart for patient selection of the SEER database. [file Image_1.JPEG]

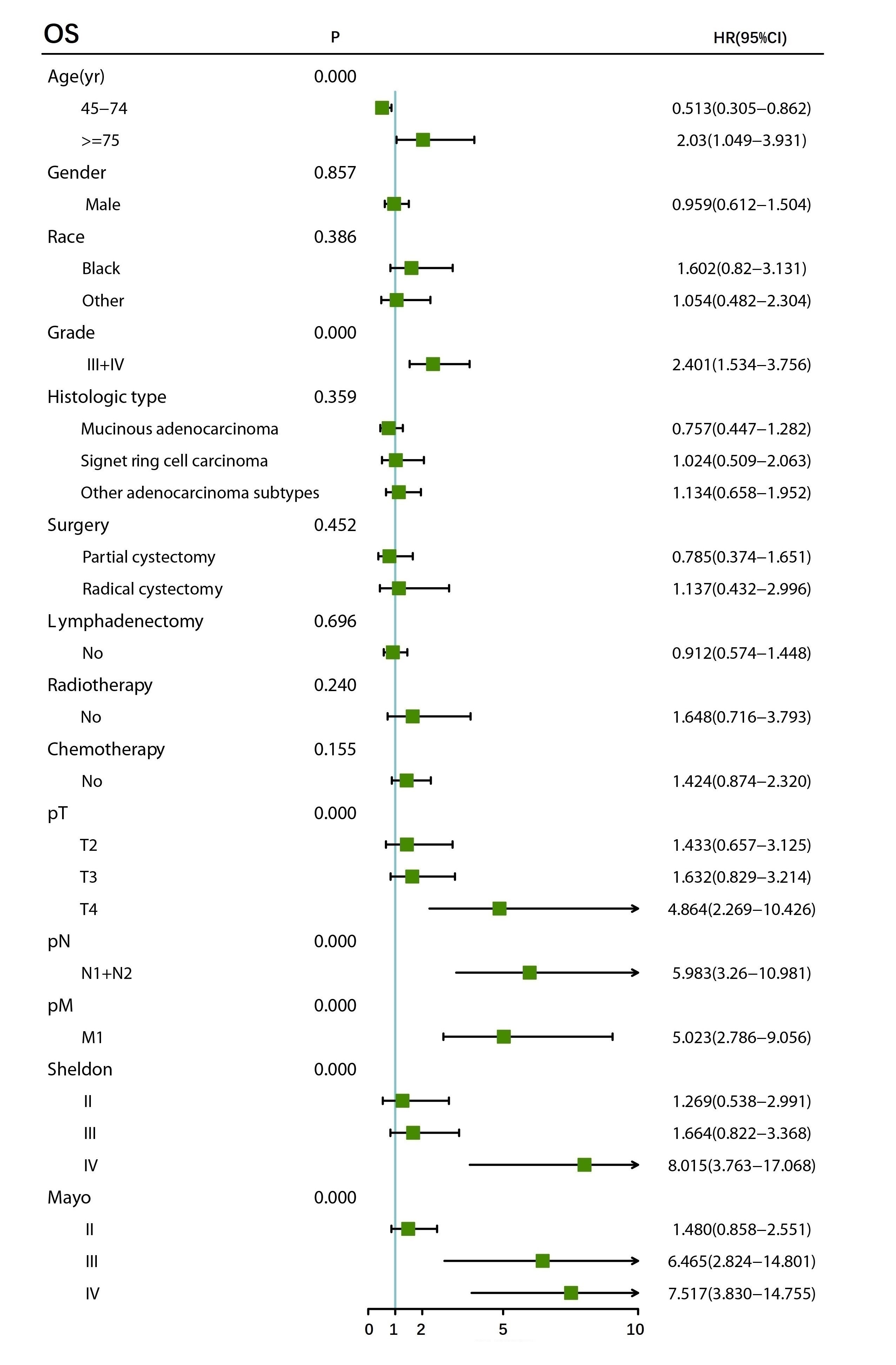

Supplement: Supplementary Figure S2 — The Forest plot of univariable Cox regression analysis of training cohort for predicting OS. [file Image_2.JPEG]

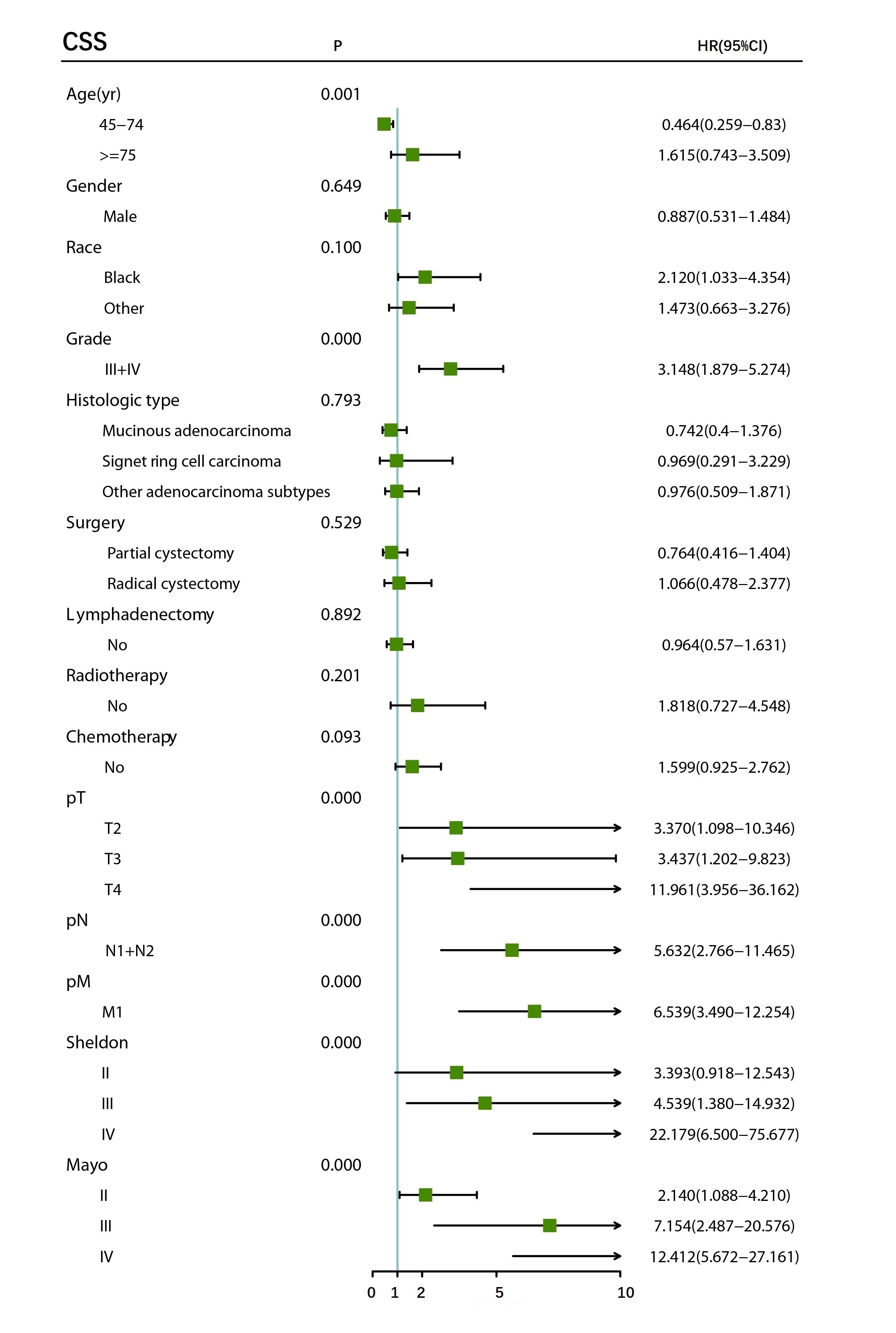

Supplement: Supplementary Figure S3 — The Forest plot of univariable Cox regression analysis of training cohort for predicting CSS. [file Image_3.JPEG]
